# Manners for Children

**Published:** 1893-02

**Authors:** 


### MANNERS FOR CHILDREN.

There are few portions of household training that are more neglected than the education of children in the habits of eating. In the family it is the easiest thing in the world to grow careless or indulge in various practices not permissible in polite society, but, all the same, these habits are formed, and the children, as a natural consequence, grow up in such ways. It is small wonder that when they find it necessary to go out into the world they are obliged to have a thorough course of training to unlearn the habits of early life.

The only excuse for this is when the parents are themselves totally ignorant of the proprieties of life. It is a poor comment on bad manners when a young person in response to reproof says: "We always did so at home." And no parent should permit it to be possible for the child to cast any such reflection on the guardian of its tender years. It is comparatively easy, once the habit of discipline is established, to compel the observance of the rules that govern good society. If parents do not know them, they should realize the necessity of learning them before they attempt the training of little children.

It must be a very unhappy reflection to father and mother when they come to comprehend the fact that their children are in disgrace because of lack of correct teaching. But this is often the case, and, though children rarely accuse the parents of being the cause of such unpleasant consequences, there are many instances where young people feel it keenly.

It is unquestionably the fact that a good deal of what is complained of by parents as neglect on the part of children comes from the feeling that they have been allowed to grow up in ignorance of many things which they should have known, and have experienced so much annoyance and discomfort on this account, that they feel sensitive and sore of spirit in consequence.

It is natural enough to feel a certain degree of resentment toward those who are the cause of serious unhappiness or social disgrace, or whether it is the parent or some one else seems to make no difference; indeed, the responsibility which attaches to that relationship but increases the discomfiture.

Social etiquette classes for the mothers of families might be a departure, but they certainly would be a lasting benefit to the rising generation.

